# Supplementary material for: Optimizing availability of obstetric surgical care in India: A cost-effectiveness analysis examining rates and access to Cesarean sections
Source: PLOS Glob Public Health. 2022 Dec 13;2(12):e0001369. doi: 10.1371/journal.pgph.0001369 (PMC10021835; doi:10.1371/journal.pgph.0001369)
Supplement: S1 Text — Fig A: (A): Markov states. (B): Decision tree for women with access to CEmOC care. (C): Decision tree for women who had no access to CEmOC care. Table A: Probabilities by number of CS. Fig B: Two-way sensitivity analysis of access to CEmOC facility and CS rates. (DOCX) [file pgph.0001369.s001.docx]

**S1 Text: Appendix.**


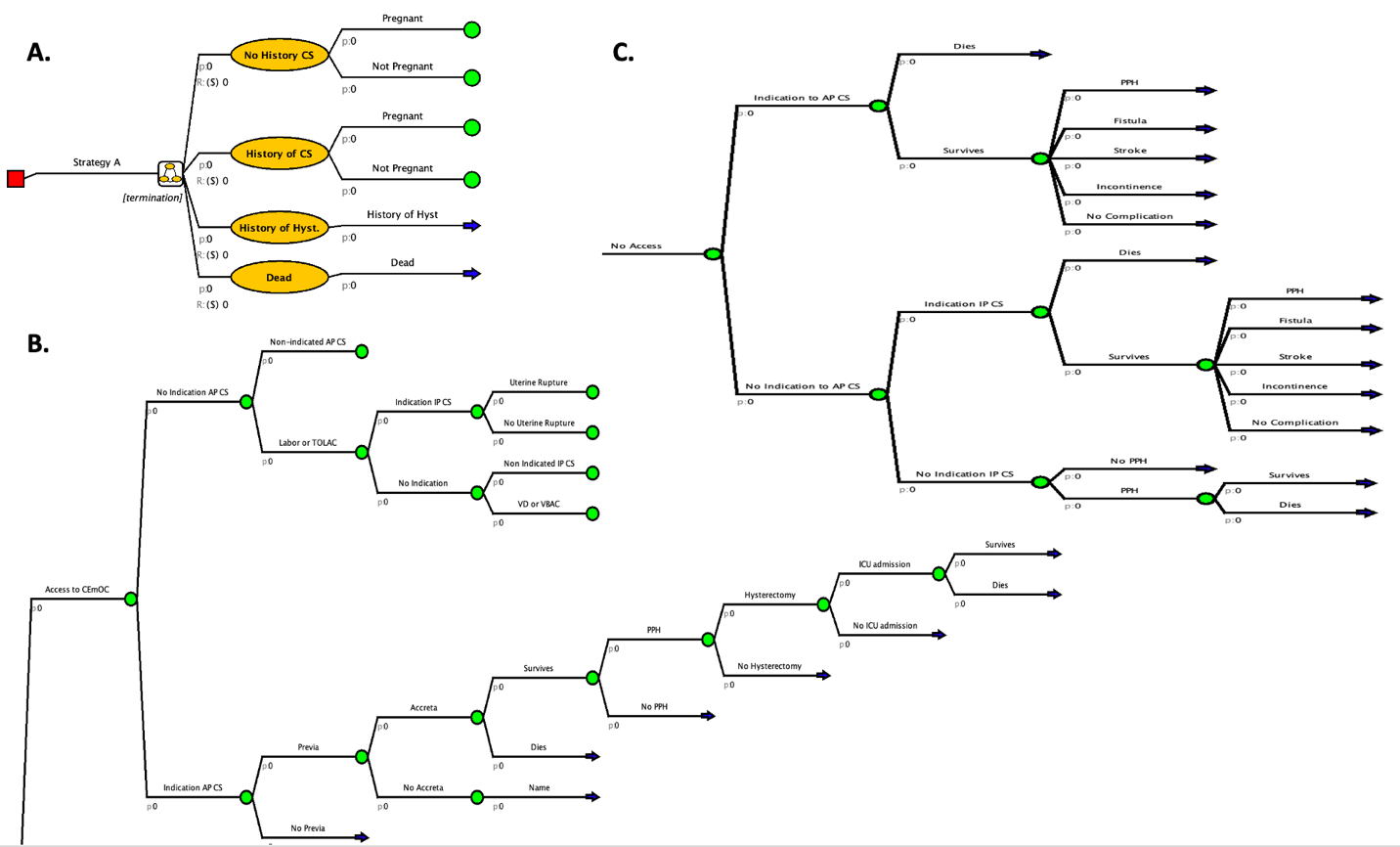


**Fig A.** (A.) The 4 Markov states for each strategy. (B.) Decision tree for pregnant women who had access to CEmOC care. (C.) Decision tree for pregnant women who had no access to CEmOC care. Note that only some representative branches are shown all the way to the terminal node. The other branches follow the same pattern. VBAC and TOLAC were branches only available for women with a history of Cesarean section. AP: Antepartum; IP: Intrapartum; PP: Postpartum; TOLAC: Trial of labor after cesarean section; VD: Vaginal delivery; VBAC: Vaginal birth after Cesarean section

| **Number of Previous CS** | **Previa** | **Accreta with Previa** | **Accreta without Previa** | **PPH with blood transfusion** |
| --- | --- | --- | --- | --- |
| **0** | .0642 | .0327 | .0003 | .0405 |
| **1** | .0133 | .1090 | .0016 | .0153 |
| **2** | .0114 | .4028 | .0011 | .0226 |
| **3** | .0227 | .6061 | .0076 | .0365 |
| **4** | .0233 | .6667 | .0078 | .0426 |
| **5 or more** | .0337 | .6667 | .0449 | .1573 |

**Table A. Probabilities by number of CS**

Probability of complications by increasing number of CS were obtained from literature of high-income countries (37).

**Fig B. Two-way sensitivity analysis of access to CEmOC facility and CS rates.**


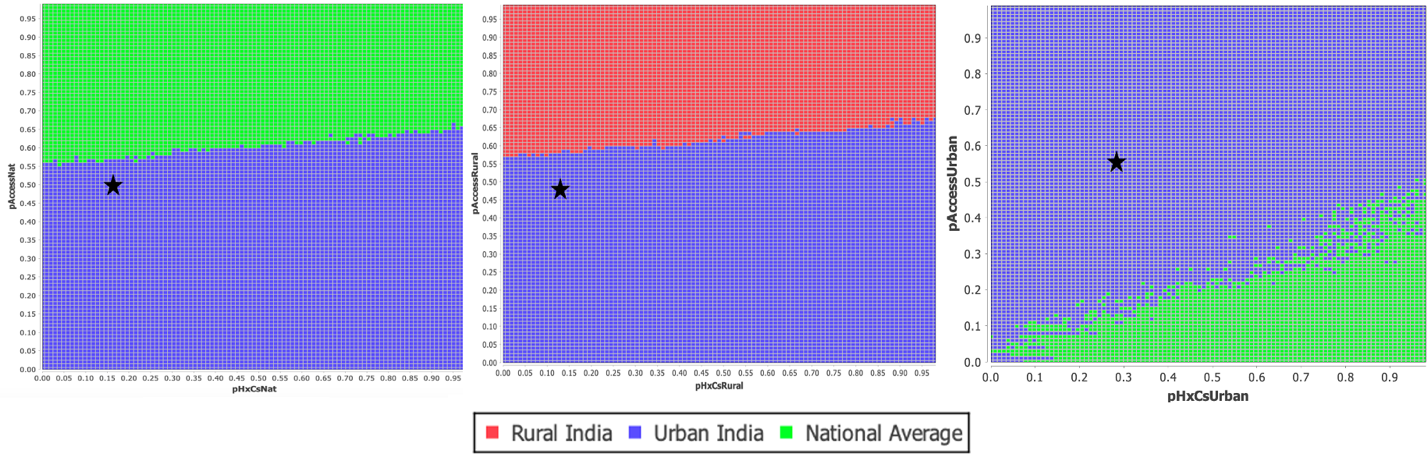


1. Strategy A (national average data) (b) Strategy B (rural data) and (c) Strategy C (urban data). Y axis indicates access to CEmOC facilities, X axis indicates history of CS and * indicates the value used in the model.
