# Supplementary material for: Optimizing availability of obstetric surgical care in India: A cost-effectiveness analysis examining rates and access to Cesarean sections
Source: PLOS Glob Public Health. 2022 Dec 13;2(12):e0001369. doi: 10.1371/journal.pgph.0001369 (PMC10021835; doi:10.1371/journal.pgph.0001369)
Supplement: Attachment — Submitted filename: Response to Reviewers Comments 13May22.docx [file pgph.0001369.s002.docx]

Dear Reviewers and editors,

Thank you very much for your time and feedback, we really appreciate it. We’ve addressed each of your comments below and have implemented your feedback which we believe has strengthened our manuscript. Please let us know if you have any further questions or concerns.

**Reviewer #1:**

**The research was rigorously done, however it was too technical and not easy to understand by a non-medical reader. In my perspective, the “strategies” used namely A, B and C where not adequately explained and very confusing. Was the national, rural and urban data used refer to as the “Strategies”, if yes, how are they strategies?**

Strategies A, B and C are hypothetical health policy strategies that assume equitable provision of cesarean sections to the entire population of India. Although these strategies are hypothetical, their components (access to CEmOC and CS rates) were based on existing data from national data, rural data and urban data. We have clarified this by updating lines 85-95:

*Optimizing CS availability thus requires a fine balance between ensuring access CEmOC facilities for all women while preventing overuse of CS without medical indication. We evaluated three hypothetical health policy strategies that assume the ability to provide equitable obstetric surgical care to the entire population of India under different assumptions. Although the strategies are hypothetical, the assumptions are based on existing national data for all of India. In Strategy A, we test a health policy strategy where the assumption is that the entire population is given access to CEoMC facilities at the current national access rate and has a CS rate equivalent to the current national average. In Strategy B, the entire population is given access to CEoMC facilities at the current rural access rate and has a CS rate equivalent to the current rural average. In Strategy C,the entire population is given access to CEoMC facilities at the current urban access rate and has a CS rate equivalent to the current urban average; Cost-effectiveness analyses (CEA) compare the costs and outcomes of different strategies and could be used to inform policies on how to scale up obstetric surgical care.* **It was also unclear as to how Strategy C was still cost-effective, notwithstanding the increased number in maternal co-morbidities and mortalities with the hypothesis stated, whereas in Strategy B (rural data), for instance with increased rates of neonatal mortality and stillbirth, it was not registered as not cost-effective.**

The main output of a cost-effectiveness analysis is the incremental cost-effectiveness ratio (ICER). We calculated the ICER for each of the three strategies (Figure 2). Usually the lower the ICER, the more cost effective an intervention will appear. Strategy B is also cost effective but Strategy C brings greater benefit (690,825 QALY in C vs 682,670 QALY in B) and is still within the willingness to pay threshold, and therefore that is the best strategy. We’ve clarified this in line 240:

*Strategy C (urban data), which extends the access and rates of CS currently observed in urban India to the entire population and has the greatest access to CEmOC facilities (55.74%) and highest CS rate (28.2%), is the cost-effective strategy (ICER is 354.90) that provides the greatest benefit (QALYs) while remaining within the WTP threshold of $1,939.61 (Figure 2). Strategy B (rural data) has an ICER of 5.20 and while cost effective, it provides less benefit (QALYs) than strategy C.*

**The term “cost-effectiveness” was not adequately contextually defined in the work as well as the term “CEmOC”. CEmOCshould clearly be define and indicates what packages or criteria of services delivery were available contextually in making the “CEmOC”. Was it just by assumption of a standard (WHO Policy) of what a CEomC facility should be? or its just for the assumption that the facility is located in an urban area? This should be clearly indicated at the introduction as this would serve as an indicator for readers.**

We’ve added the following information on cost-effectiveness analysis in the “Analysis” section (line 202):

*Cost-effectiveness analysis are policy making tools that provide a framework to assess which strategy maximizes health benefit per dollar spent. The output of a cost effective analysis that compares the strategies is the incremental cost-effectiveness ratio (ICER). We calculated the ICER for each of the three strategies, defined as the additional cost of a specific strategy divided by its additional benefit in quality-adjusted life-year (QALY), compared with the next least expensive strategy.*

We’ve added the definition of CEmOC from UNFPA followed by an explanation of how we defined CEmOC in our model and how we obtained the data to calculate access to CEmOC facilities (line 140):

*Strategies evaluated included different availability to CS depending on a combination of access to facilities that provide CS and CS rates (Figure 1). The United Nations Population Fund defines CEmOC as care typically delivered in hospitals that includes basic obstetric care plus capabilities to perform cesarean sections, blood transfusions and resuscitation of newborns. India has extensive data on access to deliveries at health care facilities but robust data on CEmOC facilities is limited to one state. Therefore access to CEmOC was defined as the probability of accessing a facility that provides an institutional delivery times the probability of accessing a facility that provides CEmOC. Data on access to institutional deliveries was obtained from national data on rural and urban areas while data on access to CEmOC facilities was available only from the state of Gujarat.*

**Reviewer #2: I commend the authors on this innovative study. I read it with interest. I have a few questions I'd appreciate their response on:
1. Is it common to use GDP per capita for the willingness to pay (WTP) threshold?**

Yes, the World Health Organization suggests that the WTP threshold be based on a country’s per capita GDP. We’ve added the following to the manuscript to clarify this (line 180):

*The willingness to pay (WTP) threshold is an estimate of what a consumer of health care is prepared to pay for the given health benefit and it is often the country’s GDP per capita. The WTP threshold we used was $1,939.61 USD, India’s GDP per capita in 2017*

**2. It's a little hard to follow the different strategies. Maybe just call them Rural Strategy, Urban Strategy, and Average Strategy?**

Thank you for the suggestion. We did not want to call the strategies rural, urban, and average as each strategy represent the hypothetical scenarios of equitable provision of CS to the entire population of India and not just the rural or urban areas. To make this clearer, after naming each strategy we have clarified in brackets if each strategy refers to national data, urban data or rural data. We have done this throughout the text and in the figures/tables.

**3. It might be helpful to mention that extrapolation of limited data means that small variations end up being magnified -- this may be why when 55.7% of the population ends up having access to surgical care, it reportedly results in MORE complications "in the next 34 years."**

Part of the reasons more complications are seen in Strategy C (urban data) is not because of increased access (55.7%) but likely because of the excessive CS rate (28.2%). With a higher number of CS rate per patient, the probability of placenta previa, accreta, hemorrhage and other complications are higher. We have clarified this in line 275: *The simulation shows an increase in the number of maternal comorbidities in strategy C (urban data) likely driven by high CS rates rather than access to surgical care. These complications are typically associated with increasing CS rates and include higher rates of placenta previa, placenta accreta, PPH, and ICU admission.*

**Is 34 years the difference between 14 year old and 49 year old? Because I'm not sure 34 is the right phrase you want there.**

It should read “35 years” instead of 34, we have fixed this error (line 282). The time horizon of 35 years was chosen as that is the definition of an average of a woman’s reproductive age (49 years-14 years). We have clarified this in line 111: *We considered all women of reproductive age (14-49 years) in India. The time horizon included a woman’s reproductive years of 35 years (49-14 years).*
